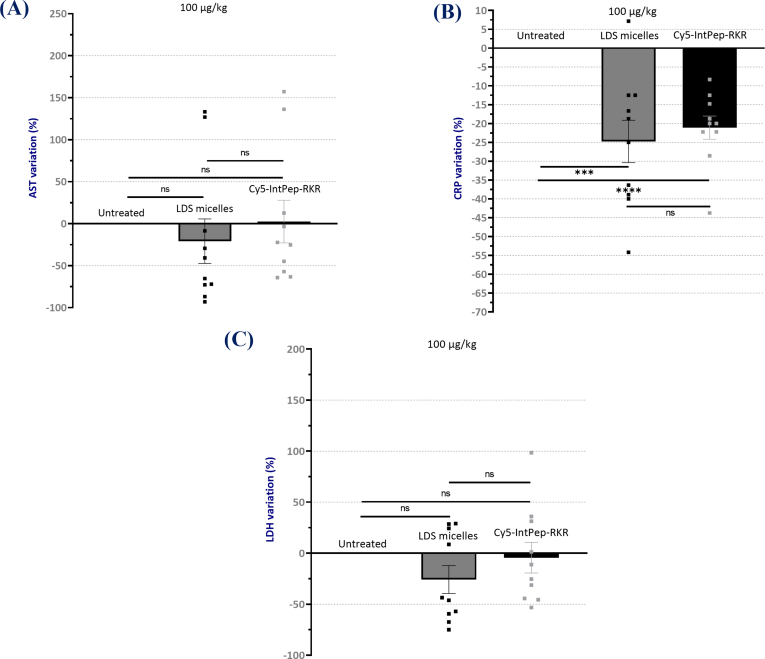# Inhibition of neuraminidase-1 sialidase activity by interfering peptides impairs insulin receptor activity *in vitro* and glucose homeostasis *in vivo*

**DOI:** 10.1016/j.jbc.2024.107316

**Published:** 2024-04-23

**Authors:** Kevin Toussaint, Aline Appert-Collin, Laetitia Vanalderwiert, Camille Bour, Christine Terryn, Caroline Spenlé, Michaël Van Der Heyden, Mathilde Roumieux, Pascal Maurice, Béatrice Romier-Crouzet, Hervé Sartelet, Laurent Duca, Sébastien Blaise, Amar Bennasroune

**Affiliations:** 1 Université de Reims Champagne-Ardenne, CNRS, MEDyC, Reims, France; 2 Université de Reims Champagne-Ardenne, PICT, Reims, France; 3 UMR7242 Biotechnology and Cell Signalling, Centre National de la Recherche Scientifique, Strasbourg Drug Discovery and Development Institute (IMS), University of Strasbourg, Illkirch-Graffenstaden, France; 4 INSERM ERL-1321, CNRS UMR7242, Peptidic Biotherapy, Strasbourg, France

**Keywords:** extracellular matrix, neuraminidase-1, insulin receptor, interfering peptides, receptor activation

## Abstract

Neuraminidases (NEUs) also called sialidases are glycosidases which catalyze the removal of terminal sialic acid residues from glycoproteins, glycolipids, and oligosaccharides. Mammalian NEU-1 participates in regulation of cell surface receptors such as insulin receptor (IR), epithelial growth factor receptor, low-density lipoprotein receptor, and toll-like receptor 4. At the plasma membrane, NEU-1 can be associated with the elastin-binding protein and the carboxypeptidase protective protein/cathepsin A to constitute the elastin receptor complex. In this complex, NEU-1 is essential for elastogenesis, signal transduction through this receptor and for biological effects of the elastin-derived peptides on atherosclerosis, thrombosis, insulin resistance, nonalcoholic steatohepatitis, and cancers. This is why research teams are developing inhibitors targeting this sialidase. Previously, we developed interfering peptides to inhibit the dimerization and the activation of NEU-1. In this study, we investigated the effects of these peptides on IR activation *in vitro* and *in vivo*. Using cellular overexpression and endogenous expression models of NEU-1 and IR (COS-7 and HepG2 cells, respectively), we have shown that interfering peptides inhibit NEU-1 dimerization and sialidase activity which results in a reduction of IR phosphorylation. These results demonstrated that NEU-1 positively regulates IR phosphorylation and activation in our conditions. *In vivo*, biodistribution study showed that interfering peptides are well distributed in mice. Treatment of C57Bl/6 mice during 8 weeks with interfering peptides induces a hyperglycemic effect in our experimental conditions. Altogether, we report here that inhibition of NEU-1 sialidase activity by interfering peptides decreases IR activity *in vitro* and glucose homeostasis *in vivo*.

First highlighted in 1960 by Warren and Spearing (1) and then studied more broadly, mammalian neuraminidases (NEUs) are known to participate in various cellular processes such as degradation in lysosomes (2), exocytosis (3), differentiation (4) or apoptosis (5). Four neuraminidases, NEU-1, NEU-2, NEU-3, and NEU-4 have been described in mammals and classified according to their cellular localization and their preferential substrate (6). NEUs also called sialidases are enzymes endowed with glycosidase activity which allows them to cleave terminal sialic acid residues from glycoproteins, glycolipids, and oligosaccharides (7). Although their characteristics are well described in the literature, more and more studies have shown that these sialidases, and in particular NEU-1, also play a role in the functioning and activation of cell surface receptors as insulin receptor (IR), epithelial growth factor receptor, low-density lipoprotein receptor and toll-like receptor 4 (8, 9, 10, 11).

NEU-1, first described as being localized in lysosomes (12), is also known to be expressed at the plasma membrane and to regulate by desialylation numerous membrane receptors such as IR (8, 13, 14). At the plasma membrane, NEU-1 can belong to the elastin receptor complex (ERC). ERC is composed of three proteins: the elastin-binding protein interacting with elastin-derived peptides (EDP) and tropoelastin, the carboxypeptidase protective protein/cathepsin A (PPCA) providing the integrity of the complex and NEU-1, the catalytic subunit (15, 16, 17). In this complex, NEU-1 has a fundamental role in (i) elastogenesis, (ii) signal transduction through this receptor, and (iii) for the EDP biological effects on atherosclerosis (18, 19, 20), thrombosis (20), insulin resistance (13), nonalcoholic steatohepatitis (21) and cancers (22, 23, 24, 25). Involvement of ERC and more precisely of NEU-1 in several physiopathologic contexts leads research teams to develop inhibitors targeting this sialidase. At present, no commercially available molecule which selectively inhibits NEU-1 exists because of the limited data about the structure of this sialidase. That’s why the function of NEU-1 is essentially analyzed by means of the broad-spectrum sialidase inhibitor N-acetyl-2,3-dehydro-2-deoxyneuraminic acid (DANA), or inhibitors of bacterial or viral NEUs, such as zanamivir or oseltamivir. All these inhibitors have unfortunately weak activity against human NEUs (26, 27). Moreover, oseltamivir induces major side effects especially in the liver (28). However, changes of the DANA scaffold have led to the development of two selective inhibitors specific of human NEU-1, the C5-hexanamido-C9-acetamido-DANA (29) and the C9-amido analog of DANA (30). Furthermore, new strategies are currently in progress to identify new promising selective inhibitors of NEU-1 (31, 32, 33, 34). Recently, our team has developed a noteworthy strategy using interfering peptides (IntPeps) to inhibit the dimerization and the activation of NEU-1 (31, 32).

IR is a tyrosine kinase receptor present at the cell surface. Binding of insulin, its main ligand, induces conformation changes of IR that leads to the transautophosphorylation of IR intracellular kinase domain at residues Y1158, Y1162, and Y1163 (35). Activation of IR and its intracellular signaling pathways such as the IRS1/PI3K/Akt (36) or RAS/extracellular signal-regulated kinase (ERK) (37) pathways allowed the use and storage of glucose by the establishment of various processes such as glucose transport, lipogenesis, or glycogenesis mainly in insulin target tissues (the liver, the white adipose tissue or the skeletal muscle) (38, 39). Many diseases are caused by a dysfunction in IR activation, and notably type 2 diabetes, characterized by the progressive development of insulin resistance in the various tissues mentioned above (39).

Emerging data suggest a strong link between NEU-1 and IR activity even if the role of NEU-1 on IR activation remains to be clarified (8, 13, 14). Indeed, under normal diet and during vascular aging associated with elastin degradation into EDP, NEU-1 plays a key role in the development of insulin resistance in mice model (13). NEU-1 inhibition by using ERC inhibitors such as DANA or chondroitin sulfate delays insulin resistance development (13). On the contrary, in an independent context of EDP and ERC activation, NEU-1 reduces insulin resistance in mice fed with high fat diet. In such a case, NEU-1 inhibition could lead to insulin resistance development (8, 14). However, data are lacking concerning the effects of specific NEU-1 inhibition on IR activity in basal condition.

In this study, we investigated the effects of NEU-1 inhibition using IntPeps on IR activation *in vitro* and *in vivo*. Using cellular overexpression and endogenous expression models of NEU-1 and IR, we have shown that IntPeps inhibit NEU-1 dimerization and sialidase activity which results in a reduction of IR phosphorylation. *In vivo*, our results showed that IntPeps are distributed in many organs and induce hyperglycemic effect in C57Bl/6J mice in our experimental conditions.

## Results

Solubilization of peptides in lithium dodecyl sulfate (LDS) micelles has been chosen to efficiently target NEU-1 at cell membrane as described by Albrecht *et al.* (31).

### Effect of IntPeps on HepG2 and COS-7 cell viability

Effects of IntPeps and LDS micelles on cell viability have been studied on HepG2 cells (Fig. S1, *A* and *B*) and on COS-7 cells overexpressing IR (Fig. S1, *C* and *D*). The concentration of 0.1 μM and two incubation times, 3 h (Fig. S1, *A* and *C*) and 24 h (Fig. S1, *B* and *D*) have been tested. These incubation times have been chosen to study the effects of a short and a long exposure time with peptides on cell viability. The choice of this concentration was based on the previous study by Albrecht *et al.* (31).

Our results show that treatment with LDS micelles or LDS micelles containing peptides (mutIntPep-RKR, the control peptide with quadruple mutation or IntPep-RKR) do not induce cell toxicity compared to the control (without treatment) (Fig. S1). We decided to use peptides at a concentration of 0.1 μM for this study. Indeed, this concentration does not induce any cell death at the several incubation times tested in this experiment, and this concentration has also been used in a previous study (31). Moreover, dynamic light scattering experiments were also used to identify the size of empty micelles or micelles containing IntPep-RKR, mutIntPep-RKR, or FITC-IntPep-RKR peptides. The results show that the different micelles have a similar size (between 2.43 and 2.92 nm) which demonstrate that the micelles are homogeneous in size (Fig. S2).

### Evaluation of IR and NEU-1 expression in HepG2 and COS-7 cells

**Figure 1:**
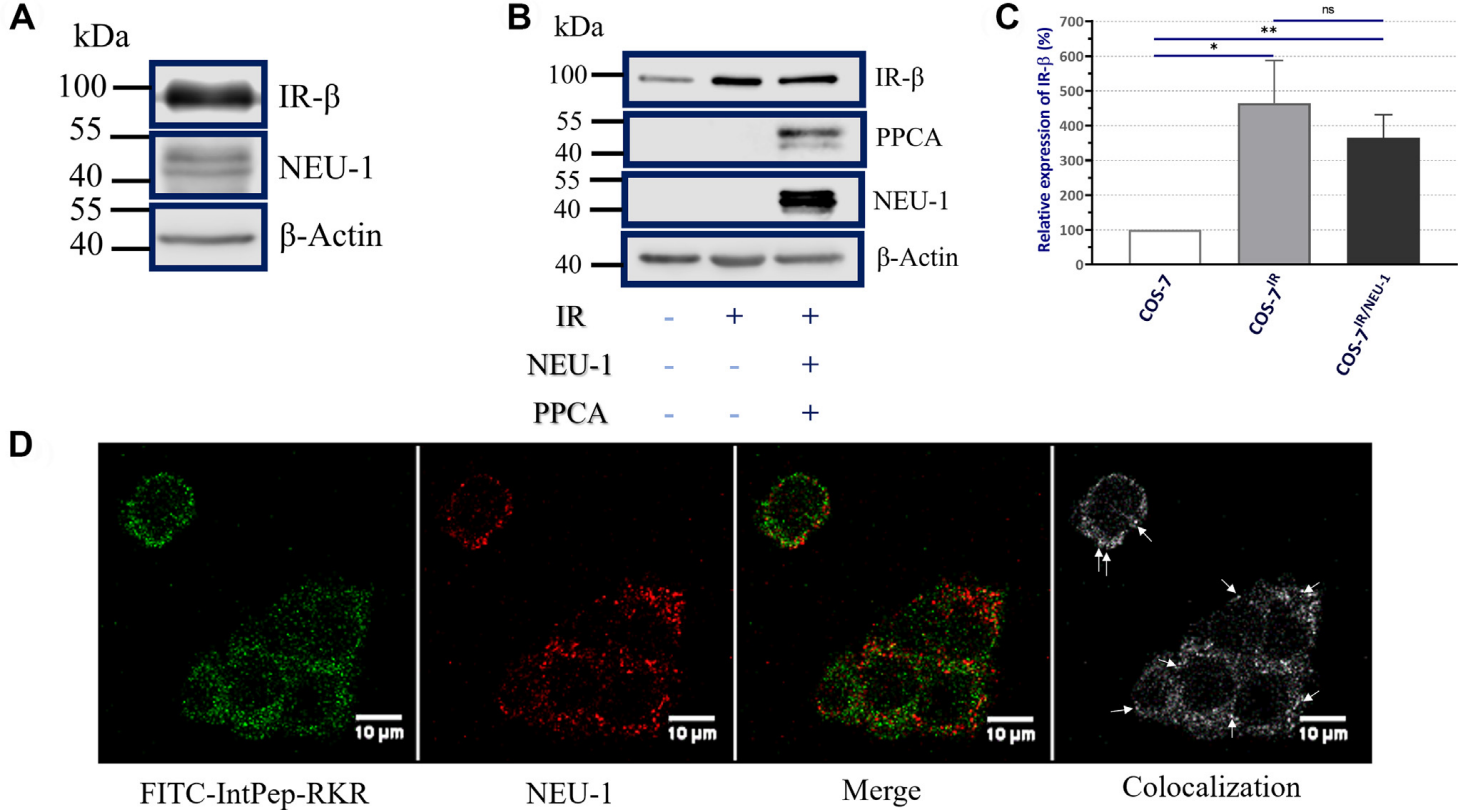
**Evaluation of IR-β, NEU-1 and PPCA expression and colocalization between interfering peptides and membrane NEU-1**. *A*, endogenous IR and NEU-1 membrane expression in HepG2 cells (n = 3). *B*, COS-7 cell line profile expression in total lysate before transfection (lane 1), after stable transfection with plasmid encoding for IR (lanes 2 and 3) and transient transfection with plasmids encoding for PPCA and NEU-1 (lane 3) (n = 4). *C*, relative expression of IR as normalized with actin in COS-7, COS-7^IR^ and COS-7^IR/NEU-1^ cells. Results are normalized using the level of IR expression in COS-7 cells. *D*, localization of FITC-IntPep-RKR at 0.1 μm (*green*) and NEU-1 (*red*) in HepG2 cells. Colocalization between interfering peptides and NEU-1 are showed by *white arrows* (*right panel*) (n = 3). IntPep, interfering peptide; IR, insulin receptor; NEU, neuraminidase; PPCA, protective protein/cathepsin A.

In the present work, two distinct cell models have been used to study the effects of IntPeps on IR activation. The human hepatocarcinoma cell line HepG2 endogenously expresses IR and NEU-1 as confirmed in Figure 1*A* and Fig. S3*A* whereas African green monkey kidney cell line COS-7 weakly expresses IR and does not express NEU-1 (Figs. 1, *B* and *C* and S3*B*) and PPCA (Figs. 1*B* and S3*B*). To study the effect of the peptides on NEU-1 and IR activation, COS-7 cells have been stably transfected with plasmid encoding human IR in order to overexpress this receptor: this cell line is named COS-7^IR^. The endogenous level of IR was assessed by immunoblotting (Fig. 1*B*). Then, transitory transfection using plasmids coding for NEU-1 and PPCA (1:2) has been made to get COS-7 cells overexpressing IR and NEU-1 (Fig. 1*B*). This cell line is named COS-7^IR/NEU-1^. Stable IR overexpression in COS-7 cells transfected or not with plasmids encoding NEU-1 and PPCA resulted in a significant upregulation of IR (by a factor of around 4) as compared with WT COS-7 cells (Fig. 1*C*).

### Colocalization between IntPeps and membrane NEU-1

The capacity of IntPeps to colocalize with NEU-1 in HepG2 cells was demonstrated by confocal microscopy (Figs. 1*D* and S4). IntPeps coupled with FITC and solubilized in LDS micelles have been incubated at 0.1 μM for 1 h with fixed HepG2 cells. The results showed a membrane localization of fluorescent peptides and NEU-1 in all observed cells (Figs. 1*D* and S4). Furthermore, our results clearly showed colocalization areas at the plasma membrane between fluorescent IntPep and NEU-1 (white arrows, Figs. 1*D* and S4) in HepG2 cells. The results show that no fluorescent labeling is visualized in HepG2 cells (Fig. S5) with FITC alone which demonstrates that the labeling obtained with FITC-IntPep-RKR micelles is not due to an integration of FITC in cell membranes.

### Effects of IntPeps on the formation of membrane NEU-1 dimers

**Figure 2:**
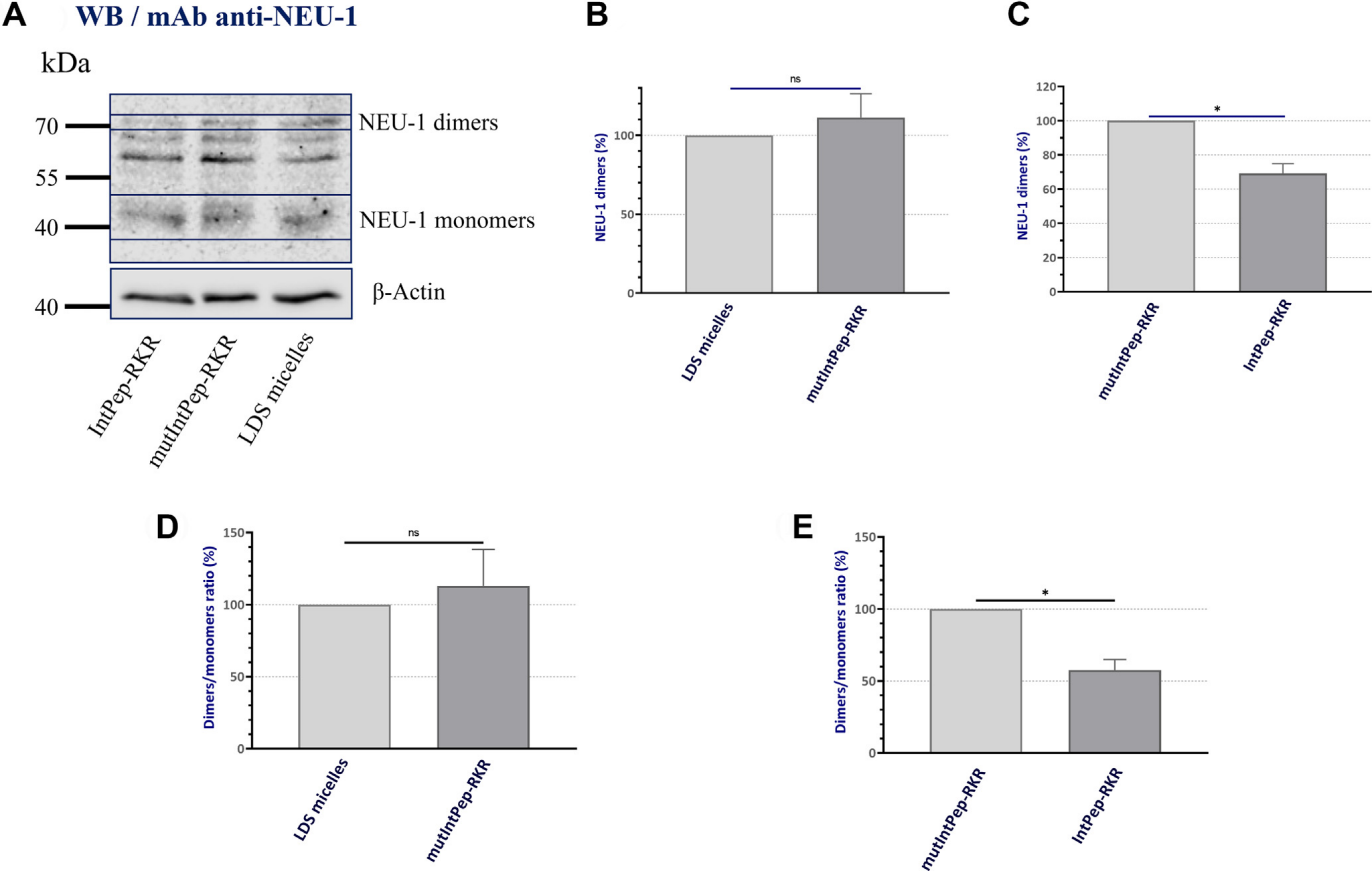
**Effects of interfering peptides on NEU-1 dimerization in HepG2 cells.***A*, Western blot showing effects of mutIntPep-RKR and IntPepRKR at 0,1 μM on NEU-1 dimerization. *B*, relative expression of NEU-1 dimers in presence of LDS micelles or mutIntPep-RKR at 0,1 μM. Results are normalized using the LDS micelle condition. *C*, relative expression of NEU-1 dimers in presence of mutIntPep-RKR or IntPepRKR at 0.1 μM. *D* and *E*, ratio of NEU-1 dimers/NEU-1 monomers expression in presence of LDS micelles, mutIntPep-RKR or IntPep-RKR at 0.1 μM. Results are normalized using the LDS micelle condition (*D*) or the mutIntPep-RKR condition (*E*) (n = 4; mean ± SEM; ns: nonsignificant; ∗*p* < 0.05; *t* test). IntPep, interfering peptide; LDS, lithium dodecyl sulfate; NEU, neuraminidase.

To evaluate the ability of IntPeps to inhibit NEU-1 dimer formation, HepG2 cell membrane extracts have been incubated with IntPeps and the effects of these peptides on NEU-1 dimer formation have been studied (Figs. 2*A* and S6). As previously described (31, 40), monomeric form (45–48 kDa) and dimeric form (70–72 kDa) of NEU-1 can be observed. NEU-1 dimer formation was not modified by mutIntPep-RKR (the control peptide with quadruple mutation) in comparison with micelles alone (Figs. 2, *A* and *B* and S6). However, IntPep-RKR decreased by 30.7% ± 5.6% the formation of NEU-1 dimers compared to the mutIntPep-RKR (Figs. 2, *A* and *C* and S6). Moreover, no difference between the ratios NEU-1 dimers/NEU-1 monomers in HepG2 cell membrane was observed when treatments are realized with LDS micelles and mutIntPep-RKR (Fig. 2*D*). However, when HepG2 cell membranes are incubated with IntPepRKR at 0.1 μM, the results show that there is a decrease of the ratio between the level expression of NEU-1 dimers and NEU-1 monomers of 42.4% ± 7.3% (Fig. 2*E*). Furthermore, two other bands comprised between 55 and 70 kDa can be observed and could correspond to hyperglycosylated monomeric NEU-1 forms as described previously (40, 41). Thus, these results demonstrate that IntPeps can specifically inhibit NEU-1 dimerization at 0.1 μM in HepG2 cells.

### Effects of IntPeps on membrane NEU-1 sialidase activity

**Figure 3:**
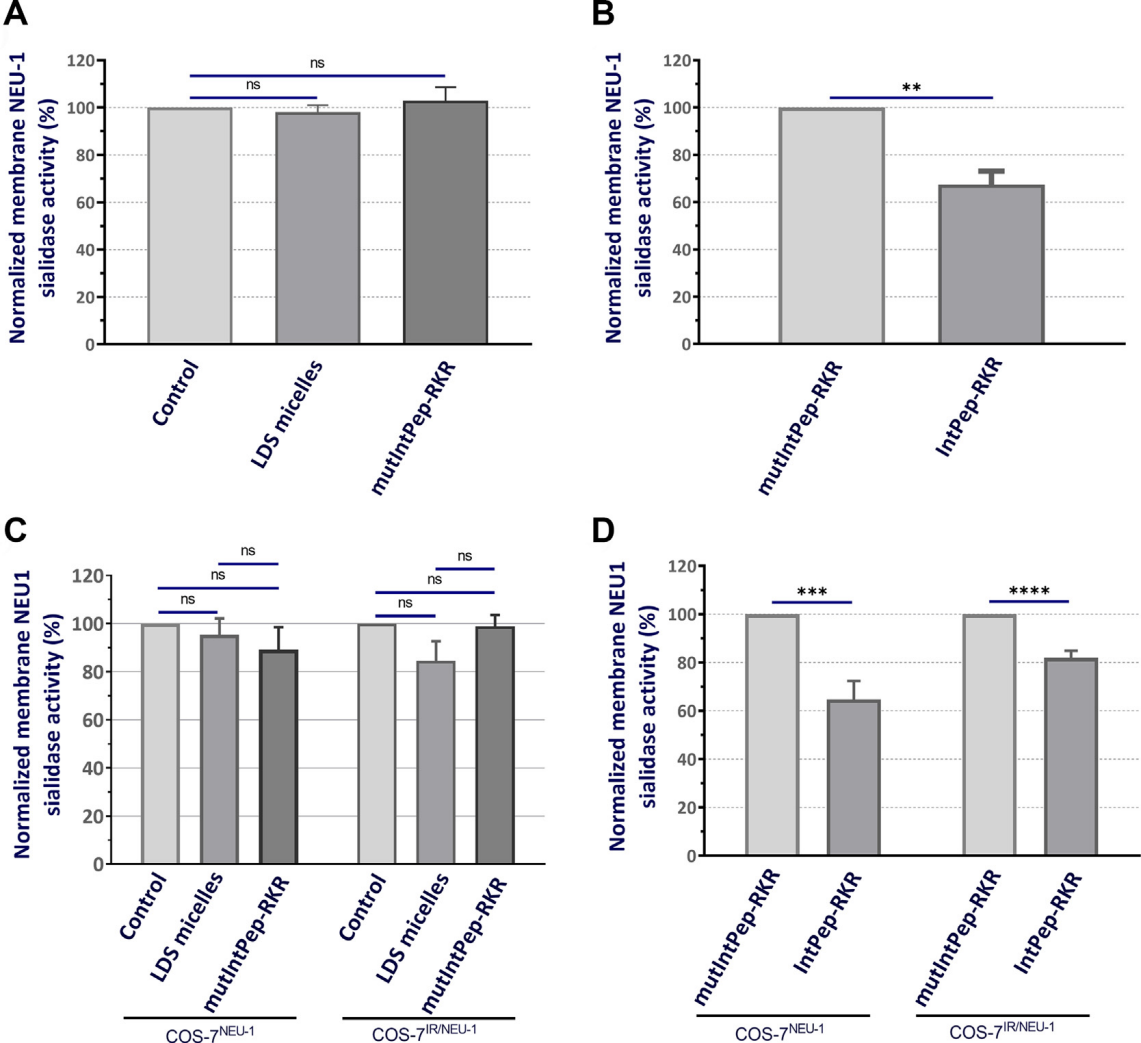
**Effects of interfering peptides on membrane NEU-1 sialidase activity in HepG2 cells and COS-7 cells overexpressing IR and NEU-1 (COS-7**^**IR/NEU-1**^**) or NEU-1 alone (COS-7**^**NEU-1**^**).***A*, normalized membrane NEU-1 sialidase activity after 15 min of incubation with LDS micelles or mutIntPep-RKR at 0.1 μM in HepG2 cells. Results are normalized using the control condition (without LDS micelle and without peptide). *B*, normalized membrane NEU-1 sialidase activity after 15 min of incubation with mutIntPep-RKR or IntPep-RKR at 0.1 μM in HepG2 cells. Results are normalized using the mutIntPep-RKR condition. *C*, normalized membrane NEU-1 sialidase activity after 15 min of incubation with LDS micelles or mutIntPep-RKR at 0.1 μM in COS-7 overexpressing NEU-1 (COS-7^NEU-1^) or IR and NEU-1 (COS-7^IR/NEU-1^). Results are normalized using the control condition (without LDS micelles and without peptides). *D*, normalized membrane NEU-1 sialidase activity after 15 min of incubation with mutIntPep-RKR or IntPep-RKR at 0,1 μM in COS-7 overexpressing NEU-1 (COS-7^NEU-1^) or IR and NEU-1 (COS-7^IR/NEU-1^). Results are normalized using the mutIntPep-RKR condition. (n = 4–5; mean ± SEM; ns: nonsignificant; ∗∗*p* < 0.01; ∗∗∗*p* < 0.001; ∗∗∗∗*p* < 0.0001; *t* test). IR, insulin receptor; LDS, lithium dodecyl sulfate; NEU, neuraminidase.

**Figure 4:**
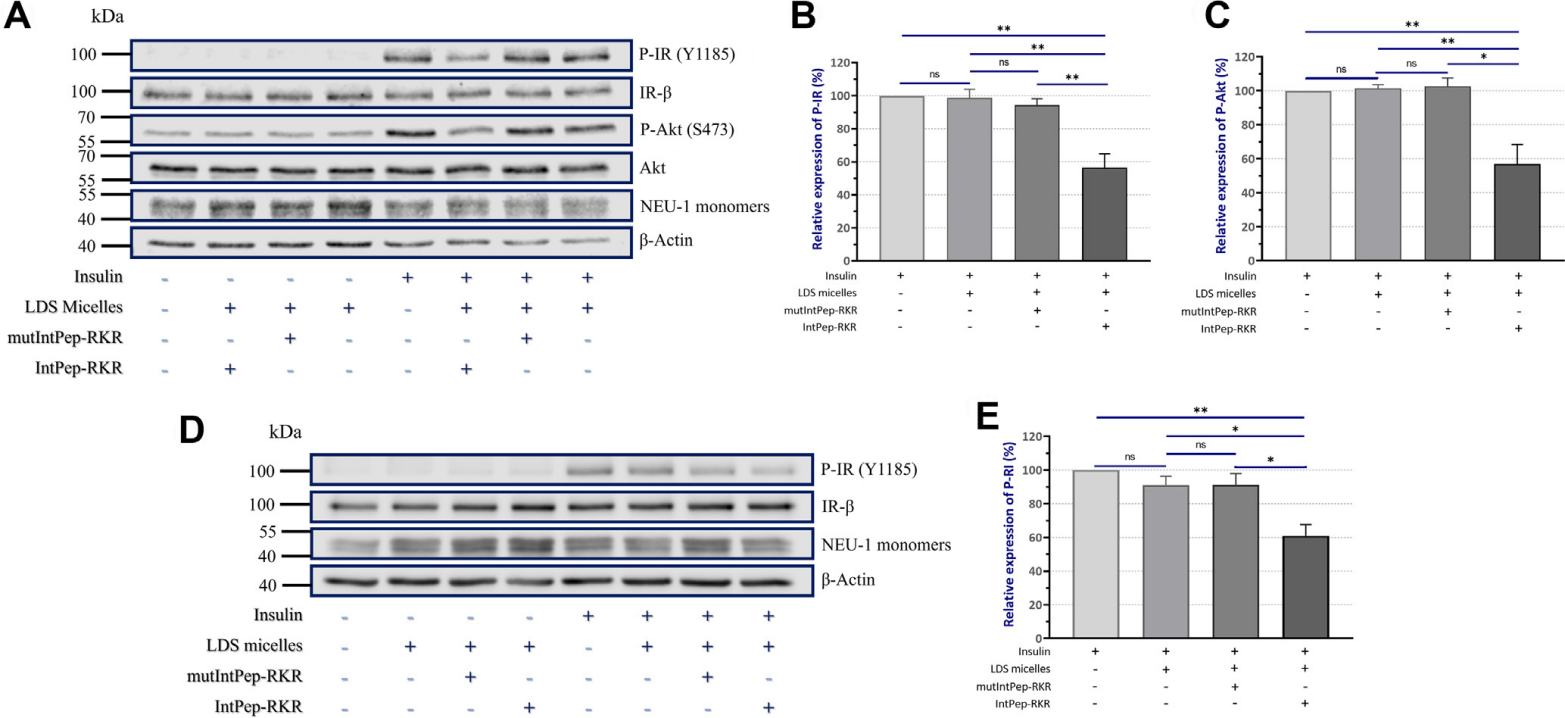
**Effects of interfering peptides on IR and Akt phosphorylation in HepG2 cells and on IR phosphorylation in COS-7 cells overexpressing IR and NEU-1.** Cells were incubated with IntPep-RKR, mutIntPep-RKR, or LDS micelles for 15 min. Insulin was added in the medium at 100 nM for 30 min before protein extraction. IR (Y1185) and Akt (S473) phosphorylation in HepG2 cells (*A*) and IR phosphorylation (Y1185) in COS-7 cells overexpressing IR and NEU-1 (COS-7^IR/NEU-1^) (*D*). Bar graphics represent the relative expression of P-IR (Y1185) and P-Akt (S473) normalized using the control condition (insulin alone) in HepG2 cells (*B* and *C*) (n = 4) and the relative expression of P-IR (Y1185) in COS-7 overexpressing IR and NEU-1 (COS-7^IR/NEU-1^) (*E*) (n = 4, mean ± SEM, ∗*p* < 0.05, ∗∗*p* < 0.01, ns: nonsignificant, *t* test). IR, insulin receptor; LDS, lithium dodecyl sulfate; NEU, neuraminidase.

Given that NEU-1 sialidase activity is linked to its ability to dimerize, we studied effects of IntPeps on NEU-1 sialidase activity in HepG2 cells. The effects of peptides on sialidase activity were quantified after 15 min of incubation with crude membrane. The mutIntPep-RKR at 0.1 μM did not modify NEU-1 sialidase activity compared to the control (Fig. 3*A*). Same results were obtained on COS-7 overexpressing NEU-1 (COS-7^NEU-1^) or IR and NEU-1 (COS-7^IR/NEU-1^) in the same conditions (Fig. 3*C*). On the contrary, IntPep-RKR at 0.1 μM decreased membrane NEU-1 sialidase activity by 32.6% ± 5.7% compared to mutIntPep-RKR used at the same concentration in HepG2 cells (Fig. 3*B*). Moreover, IntPep-RKR at 0.1 μM significatively decreased membrane NEU-1 sialidase activity by 35.3% ± 7.6% in COS-7^NEU-1^ and by 18.1% ± 2.9% in COS-7^IR/NEU-1^ compared to mutIntPep-RKR used at the same concentration (Fig. 4*D*). Taken together, these results showed that IntPep-RKR can inhibit membrane NEU-1 sialidase activity in HepG2 cells and COS-7 cells overexpressing NEU-1 or IR and NEU-1.

### Effects of IntPeps on IR phosphorylation

**Supporting Figure S8:**
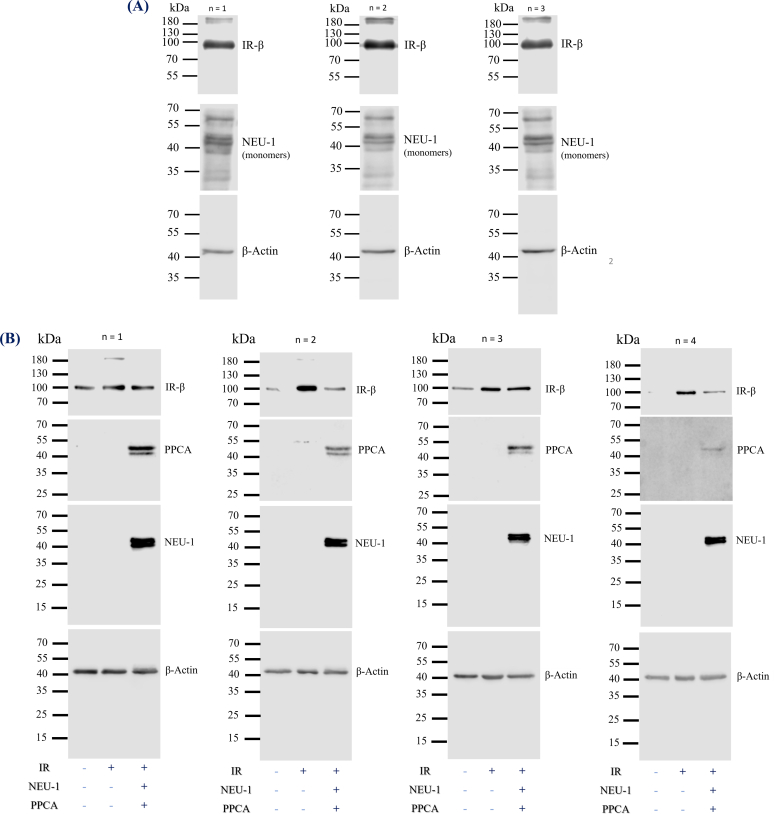


To evaluate the ability of IntPeps to inhibit IR phosphorylation, HepG2 cells and COS-7^IR^ cells overexpressing NEU-1 were first incubated with IntPeps (0.1 μM) or micelles for 15 min and then with insulin at 100 nM for 30 min. IR phosphorylation was studied on whole cell extracts by Western blotting. The results showed that LDS micelles do not modify IR phosphorylation on Y1185 residue and Akt phosphorylation on S473 residue induced by insulin compared to a treatment of HepG2 cells by insulin alone (Figs. 4, *A*–*C* and S7). The same results on IR phosphorylation are observed in COS-7^IR/NEU-1^ cells (Figs. 4, *D* and *E* and S8). In addition, no modification of the level of IR (Y1185) and Akt (S473) phosphorylation induced by insulin in HepG2 cells exists between LDS micelles and mutIntPep-RKR conditions (Figs. 4, *A*–*C* and S7); the same results are obtained in COS-7^IR/NEU-1^ cells on IR phosphorylation (Figs. 4, *D* and *E* and S8). However, a significant decrease of IR phosphorylation of 39.6% ± 9.6% and Akt phosphorylation of 42.4 ± 7.3% has been observed with IntPep-RKR compared to mutIntPep-RKR condition in HepG2 cells (Figs. 4, *B* and *C* and S7). Concerning COS-7^IR/NEU-1^ cells overexpressing NEU-1, we also observed a significant reduction of IR phosphorylation of 32.3% ± 7.5% with IntPep-RKR compared to mutIntPep-RKR peptides (Figs. 4*D* and S8). Thus, IntPeps inhibit IR transautophosphorylation on Y1185 which suggests that NEU-1 inhibition leads to an inhibition of IR and Akt phosphorylation and activation. Hence, our results suggest that in our experimental conditions, NEU-1 positively regulates IR phosphorylation and activation in accordance with the studies conducted by Dridi *et al.* (14).

### Systemic and cellular toxicity of IntPeps in C57Bl6J mice

The systemic and cellular toxicity of IntPeps in C57Bl/6J mice (twice injection per week at 100 μg/kg for 8 weeks) have been checked in plasma samples by quantification of metabolic enzymes and expression level of an inflammatory marker, the C-reactive protein (CRP) (Fig. S9). No significant difference between the two groups (treated with LDS micelles or cyanine-5 (Cy5)-IntPep-RKR) were found in the liver enzyme activity as aspartate transaminase (AST) (Fig. S9*A*), expression level of CRP and the cellular stress enzyme activity, lactate dehydrogenase (LDH), (Fig. S9, *B* and *C*). No significant difference of AST and LDH activity levels was observed between the two groups of mice after treatment (Fig. S9, *A* and *C*). However, we have highlighted a decrease of CRP level in LDS micelle (24.77% ± 5.6%) and Cy5-IntPep-RKR (21.11% ± 3.1%) groups by comparing data before and after treatment (Fig. S9*B*). Many parameters can influence these significant differences like the quality of our plasma samples or the animal stress during experiment. Nevertheless, the applied treatment did not induce significant increases in AST or LDH activities and in level of CRP which indicate that there is no systemic or cellular toxicity and no inflammatory reaction. Thus, taken together, the 8-week treatment corresponding to two injections per week of LDS micelles or Cy5-IntPep-RKR at 100 μg/kg did not induce toxicity and inflammation in mouse model. These overall data suggest that the experimental conditions selected for this study (dose and frequency for LDS micelles and Cy5-IntPep-RKR injections) can be used for studies on C57Bl/6J mice.

### Biodistribution of IntPeps in C57Bl/6J mice

**Figure 5:**
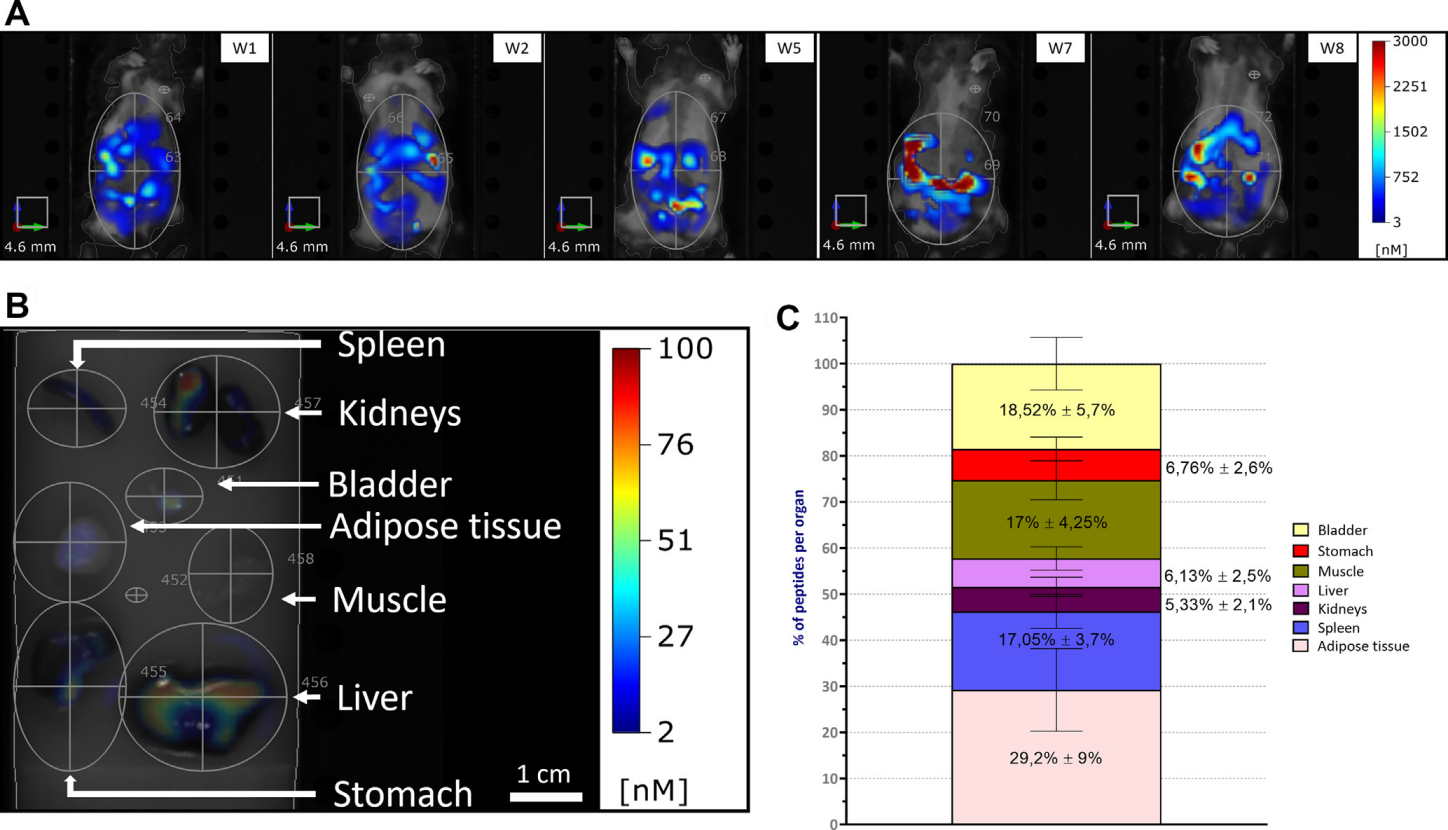
**Biodistribution of TM peptides during 8 weeks of treatment in C57Bl6J mice.** Peptide injections are performed intraperitoneally twice per week and at 100 μg/kg. The biodistribution has been measured each week in entire body (*A*) and organ per organ (*B*) at the end of the 8 weeks of treatment in C57Bl6J mice. *C*, graphic represents the peptide amount proportion found into each studied organ (*beige*: adipose tissue, *blue*: spleen, *violet*: kidneys, *pink*: liver, *green*: muscle, *red*: stomach, *yellow*: bladder) (n = 10; mean ± SEM). TM, transmembrane domain.

Biodistribution of IntPeps coupled with cyanine-5 (Cy5-IntPep-RKR) in C57Bl/6J mice was studied to learn more about the behavior of these peptides in whole organism during chronic treatment. Cy5-IntPep-RKR and LDS micelles were injected intraperitoneally twice per week at 100 μg/kg for 8 weeks. The fluorescence in the whole body was measured five times during the treatment and 15 min after injection of Cy5-IntPep-RKR (Fig. 5, *A* and *B*). We can notice that fluorescence is well distributed in the whole organism in mice during the weeks 1,2,5,7, and 8 (Fig. 5*A*). Indeed, fluorescence is present in digestive system region, in the liver or in urinary system region throughout the treatment. To determine more precisely the biodistribution of the peptides, the fluorescence has been measured and quantified organ per organ in mice after 8 weeks of treatment (Fig. 5, *B* and *C*). Autofluorescence of organs has been determined from the level of fluorescence measured in organs of C57Bl/6J mice receiving LDS micelles. Cy5-IntPep-RKR is distributed in all organs selected for this study and especially in the white adipose tissue (29.2% ± 9%). Moreover, fluorescent peptides are also found in two other organs that become insulin resistant in diabetic mice: the muscle (17 ± 4.25%) and the liver (6.13% ± 2.5%) (Fig. 5*C*). Finally, peptides are also found in organs involved in toxin elimination as the bladder (18.52% ± 5.7%), the spleen (17.05% ± 3.7%), and kidneys (5.33% ± 2.1%) (Fig. 5*C*). Taken together, these data show that IntPeps are well distributed in the organism (notably in the mentioned organs) after 8 weeks of treatment in C57Bl/6J mice.

### Effects of IntPeps on metabolic parameters in C57Bl/6J mice

**Figure 6:**
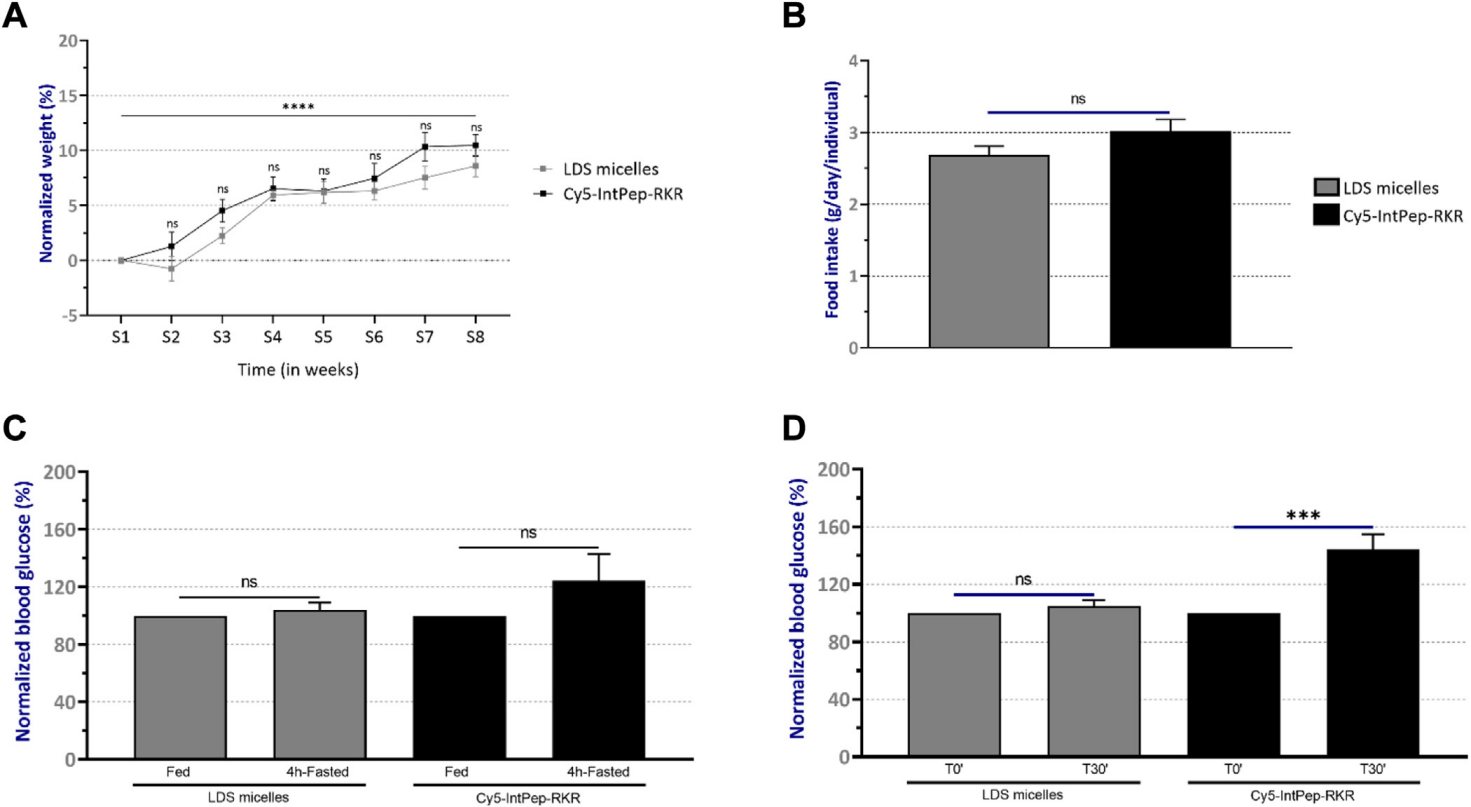
**Effects of interfering peptides on weight, food intake, and blood glucose after 8 weeks of treatment in C57Bl6J mice.** Weight is measured twice per week and normalized with the weight measured the first week of the treatment in C57Bl6J mice (*A*) (n = 10 per group). *B*, food intake studied the last week of treatment (n = 6). *C*, blood glucose level in nonfasted or 4 h-fasting C57Bl/6J mice (n = 10 per group). *D*, blood glucose level in C57Bl/6J mice without fasting before injection and 30 min after injection of interfering peptides (n = 10 per group). (Mean ± SEM, ∗∗*p* < 0.01, ∗∗∗∗*p* < 0.0001, ns: nonsignificant, *t* test).

During the 8 weeks of treatment, several parameters have been evaluated: the evolution of mice weight, the food intake and the blood glucose before and after injection of peptides at 100 μg/kg, twice per week. Our results showed that IntPeps do not induce modification of weight during 8 weeks of treatment compared to a treatment with LDS micelles in C57Bl/6J mice (Fig. 6*A*). These results are consistent with those concerning food intake (Fig. 6*B*) since no difference of eating behavior between the two groups was observed. Indeed, IntPeps do not impair the food intake after chronic treatment of 8 weeks (Fig. 6*B*). Then we measured blood glucose in mouse groups fasted for 4 h or not fasted. We observed that there is no difference of blood glucose level between the two groups when mice are fasted or fed (Fig. 6*C*). Finally, we studied the blood glucose of mice without fasting period before injection and 30 min after injection of IntPeps (or LDS micelles) (Fig. 6*D*) under isoflurane anesthesia. Our results showed that there is no effect of LDS micelles on blood glucose level in C57BL/6J mice (Fig. 6*D*). However, blood glucose of mice receiving Cy5-IntPep-RKR is increased by about 44.5% ± 10.4% after peptide injection at 100 μg/kg compared to blood glucose of control mice (before injection) (Fig. 6*D*). Taken together, these data suggest hyperglycemic effect in C57BL/6J mice after treatment with IntPeps which inhibit dimerization and sialidase activity of NEU-1.

## Discussion

For some years now, NEU-1 involvement in different cellular mechanisms has been described which allowed to consider NEU-1 as a new pharmacological target in several physiopathologic context. Indeed, it has been shown that activation of this sialidase by EDP *via* ERC is involved in the development of numerous disorders such as atherosclerosis (19), thrombosis (20), nonalcoholic steatohepatitis (21), insulin resistance (13) and cancers (22, 25).

Several broad-spectrum inhibitors for neuraminidases exist such as DANA and Tamiflu/Oseltamivir which are the most commonly used inhibitors in research. However, these inhibitors do not specifically target NEU-1. Therefore, chemical inhibitors derived from DANA such as C9-BA-DANA and C5-hexanimido-C9 acetamido-DANA which specifically target NEU-1 have been developed (29, 30). However, other inhibition strategies are necessary to elaborate molecules that specifically inhibit NEU-1 in view of the importance of this sialidase in various types of diseases. For example, recent studies allowed to identify potential inhibitors of NEU-1 from natural molecules extracted from several plants. These bioactive compounds could be used as a starting point for the development of new natural putative NEU-1 inhibitors and the design of more potent synthetic compounds (33, 34, 42). Moreover, previous studies have shown that NEU-1 possesses two segments which are potential transmembrane domains (TM1: 139–159 and TM2: 316–333) and that TM2 sequence is involved in dimerization and sialidase activity of NEU-1 (40). These results led us to develop hydrophobic IntPeps mimicking TM2 domain in order to target specifically NEU-1 by inhibiting its dimerization and its sialidase activity (31, 32). Two approaches have been tested to deliver interferent peptides: IntPep were coupled to HIV-1-TAT sequence (TAT strategy) or solubilized in detergent LDS micelles (LDS micelle strategy). The results showed that the second strategy offer more advantages than the first one. Indeed, the peptide concentrations used with LDS micelle strategy (0.1 μM) allowed to obtain levels of NEU-1 inhibitions (dimerization and sialidase activity) similar to those obtained with the TAT strategy (5 or 10 μM of peptides) with lower concentrations (31). Furthermore, it has been demonstrated in other studies that peptides solubilized in micelles adopt an optimal conformation allowing better interaction with their target and that there is a better resistance to protein degradation (43, 44). Thus, the strategy of IntPeps solubilized in LDS micelles has been chosen for this study.

Because of the involvement of NEU-1 in the development of insulin resistance, many studies have focused on the link between this sialidase and IR (8, 13, 14). In a context linked to vascular aging and elastin degradation, Duca *et al.* have shown that EDP are able to activate NEU-1 by interacting with ERC (16). In addition, the study of Blaise *et al.* suggested that NEU-1 inhibits IR phosphorylation and activation leading to the development of insulin resistance and glucose intolerance (13). However, other studies have reported an opposite effect of NEU-1 on the IR activation. For instance, in the absence of NEU-1 and under fatty diet, IR signaling is partially affected resulting in a more rapid development of insulin resistance and glucose intolerance (14). In addition, NEU-1 overexpression in insulin-resistant HepG2 cells reverses this insulin resistance. The same effect was observed after stimulation of NEU-1 activity by ambroxol in mice fed with a fat diet (8). All these data suggest that in these conditions, NEU-1 has a protective effect by promoting IR activation (8, 14).

The aim of this study was to determine the effects of a specific inhibition of NEU-1 by IntPeps on the IR activation *in vitro* and *in vivo*. Indeed, the close link between NEU-1 and IR activations has been demonstrated by different research teams as described above. However, NEU-1 effects on IR seem to be different according to the physiologic context (8, 13, 14). That is why we choose to evaluate the effect of specific inhibition of NEU-1 *in vitro* and *in vivo* by using IntPeps which specifically target this sialidase in conditions without ERC activation by EDP.

To answer these questions, we first evaluated the localization of FITC-IntPep-RKR delivered by LDS micelles and NEU-1 in cells that endogenously express NEU-1. Our results showed colocalization areas at the plasma membrane between FITC-IntPep-RKR and NEU-1 which suggest that IntPep are able to anchor in the plasma membrane and interact with NEU-1. Subsequently, we investigated the ability of IntPep to inhibit dimerization and sialidase activity of NEU-1. Significant decreases in NEU-1 dimerization (∼30.7%) and sialidase activity (∼32.6%) induced by IntPep-RKR were observed in HepG2 cells. Same results of sialidase activity were obtained for COS-7 cells overexpressing NEU-1 (∼35%) or IR and NEU1 (∼18%). However, these inhibition levels are not complete which can be explained by the presence of a second dimerization domain (named TM1) in NEU-1 (40). Even if the TM1 dimerization potential is lower than that of TM2, the putative formations of TM1/TM1 homodimers and TM1/TM2 heterodimers can explain this partial inhibition of NEU-1 obtained with IntPep-RKR. Indeed, as the interferent peptides used in this study specifically target NEU-1 TM2 domain, they cannot disturb TM1/TM1 homodimers formation and induce complete inhibition of NEU-1. Similar data were obtained in a previous study using a cell line overexpressing NEU-1 (31). Therefore, it would be interesting to evaluate the capacity of a combination of peptides corresponding to the TM1 and TM2 domains to increase the inhibition level of NEU-1 dimerization and sialidase activity in HepG2 cells and COS-7 cells overexpressing NEU-1.

Subsequently, we studied the effect of IntPeps on the IR phosphorylation and thus on its activation. We noticed a decrease in the IR phosphorylation at Y1185 (or 1158) residue involved in IR activation and in the Akt phosphorylation at S473 residue in HepG2 cells. Same data were observed for IR phosphorylation in COS-7 overexpressing IR and NEU-1. The inhibition of membrane NEU-1 by IntPep partially inhibits phosphorylation and activation of IR. Since the sialidase activity of membrane NEU-1 was not completely reduced, it is rather consistent that IR phosphorylation is also in part inhibited. These results are consistent with the study of Fougerat *et al.* which showed that desialylation of IR by NEU-1 induces the active conformation of IR dimer. Moreover, their results demonstrate that DANA (which inhibits NEU-1 activity) leads to the decrease of IR desialylation by NEU-1 which is associated with a decrease of IR activation (8).

We then examined the biodistribution of IntPep in the whole organism of a healthy C57Bl/6J mouse model during an 8-week treatment. Our data show that IntPep are distributed in the organism during the treatment period and especially in the digestive, urinary, and hepatic systems. These results were confirmed by the study of peptide distribution in several organs. IntPep were found in organs responsible for toxin elimination such as kidneys and the bladder which means that they are well eliminated. We also found IntPep in organs or tissues that are concerned by insulin resistance, that is, liver, adipose tissue, and muscle. IntPep treatment did not induce any significant weight gain or loss and did not alter the amount of food consumed by C57Bl/6J mice. Taken together, these results suggest that IntPep have no effect on weight and on eating habits of mice. Concerning the study of blood glucose levels before and after injection, our results show an increase in blood glucose in mice. Moreover, it will be interesting to study the effect of IntPep on blood glucose according to the sexes. Indeed, data in link with differences in response between males and females are emerging in many studies concerning blood glucose (45, 46, 47). For example, metabolic dysfunction (weight gain, fat depot gains, and insulin resistance development) of female C57Bl/6J mice fed with high fat diet is delayed compared to males (46). Furthermore, our results show that specific inhibition of NEU-1 by IntPep reduces IR function leading to hyperglycemia. The decrease in IR phosphorylation observed *in vitro* is consistent with the immediate hyperglycemia induced by IntPep inhibiting NEU-1 in C57Bl/6J mice. Therefore, the present work suggests that without activation of ERC by EDP, inhibition of NEU-1 negatively regulates IR function *in vitro* or *in vivo* as it has been shown in previous studies in other experimental conditions (8, 14).

**Figure 7:**
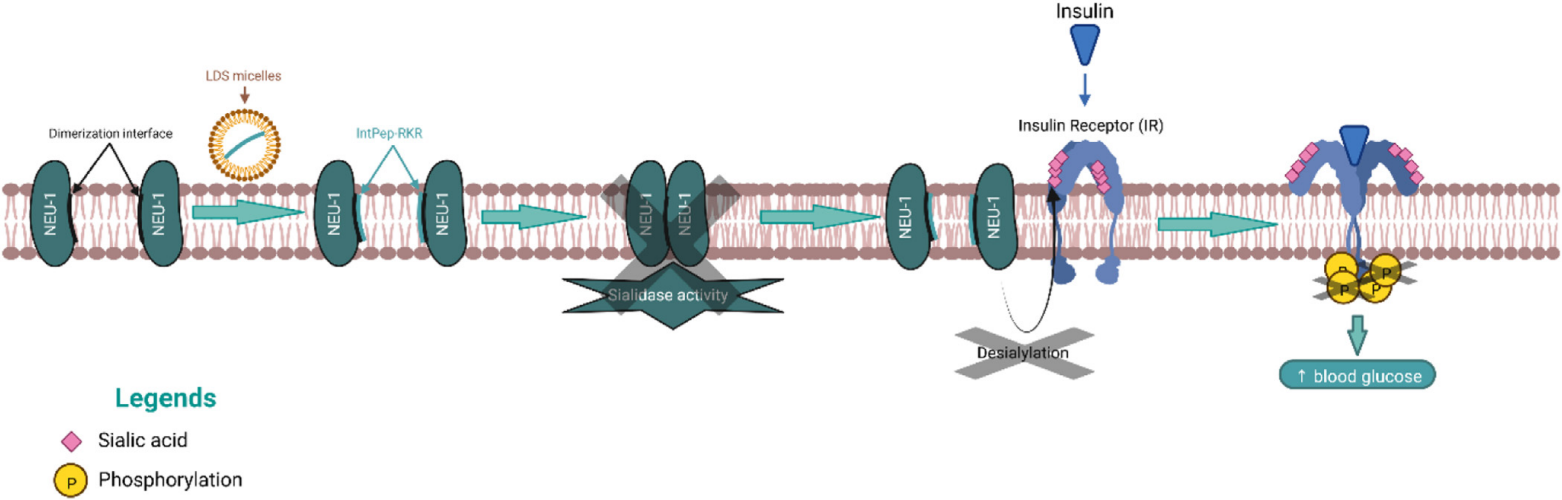
**Summary of the mechanism suggesting effects of NEU-1 inhibition on IR activation.** Interfering peptides target NEU-1 to prevent its dimerization and its sialidase activity. NEU-1-dependent IR desialylation could be therefore impaired which leads to the inhibition of IR phosphorylation and activation. This inhibition of IR activity results in an increase of blood glucose in C57Bl/6J mice. Created with BioRender.com. IR, insulin receptor; NEU, neuraminidase.

Thus, our results show that IntPep are able to specifically inhibit NEU-1 dimerization and sialidase activity in HepG2 cell line which leads to an inhibition of IR phosphorylation resulting in an increase of blood glucose in C57Bl/6J mice (Fig. 7).

In conclusion, the present work shows that inhibition of NEU-1 catalytic activity by IntPep is associated with an inhibition of IR activation. In the context of vascular aging and in particular during elastin degradation and the release of EDP, it has been shown that the development of insulin resistance is mediated by NEU-1 activity (13). Subsequently, it would be interesting to study the effects of NEU-1 specific inhibition by using IntPep on IR function using insulin resistance model as db/db mice.

## Experimental procedures

### Peptide solubilization

The different peptides were purchased from GENEPEP, and according to manufacturer’s indications, their purity determined by reversed-phase-HPLC was about 95%. The different peptide sequences are as follows: *E*_*312*_*LVDPVVAAGAVVTSSGIVFFS*_*333*_*-RKR* (IntPep-RKR), *E*_*312*_*LVDPVVVAIAVVTSSIIAFFS*_*333*_*-RKR* (mutIntPep-RKR), FITC-LC- *E*_*312*_*LVDPVVAAGAVVTSSGIVFFS*_*333*_*-RKR* (FITC-IntPep-RKR) and H-C(Cy5)-*E*_*312*_*LVDPVVAAGAVVTSSGIVFFSRKR*_*333*_*-NH2* (Cy5-IntPep-RKR). All the peptides were solubilized in LDS micelles as described by Roth *et al.* (31, 48). As described by Bennasroune *et al.*, peptides included in micelles were incubated with cells in a small volume in order to get a detergent concentration far less than its critical micelle concentration, permitting partitioning of the peptides in cell membranes (49). Micelle averaged hydrodynamic diameters (Z-ave) were determined by dynamic light scattering as described by Albrecht *et al.* (31).

### Cell cultures, transfections, and IR stimulation

Plasmid encoding human IR was a gift from Dr Tarik Issad (Institut Cochin, Paris, France) and plasmid encoding human PPCA protein was provided by Pr. Alessandra d’Azzo (St Jude Children's Research Hospital, Memphis, USA). These two plasmids have been previously described (50, 51). Plasmid encoding human NEU-1 was purchased from ImaGenes GmbH. For cell transfections, JetPEI DNA transfection reagent was purchased from Polyplus transfection. In this study, HepG2 cells (ATCC HB-8065), a human hepatocarcinoma cell line and COS-7 cells (ATCC CRL-1651), a fibroblast-like cell line derived from green African monkey kidney were used. HepG2 cells were harvested in 1 g/L glucose Dulbecco’s modified Eagle’s medium supplemented with 10% heat inactivated fetal bovine serum, 100 units/ml penicillin, 0,1 mg/ml streptomycin at 37 °C in a humidified atmosphere at 5% CO_2_. COS-7 cells were maintained in culture in 4.5 g/L glucose Dulbecco’s modified Eagle’s medium supplemented with 10% heat-inactivated fetal bovine serum, 100 units/ml penicillin, 0.1 mg/ml streptomycin at 37 °C in a humidified atmosphere at 5% CO_2_. COS-7 cells have been stably transfected with plasmid encoding human IR. Geneticin selection (400 μg/ml) has been made after transfection to obtain a stable cell line overexpressing IR named COS-7^IR^. Geneticin (80 μg/ml) were added in the completed medium to maintain this cell line. Moreover, COS-7 or COS-7^IR^ cells have been transiently transfected with PPCA and NEU-1 plasmids (2:1) using jetPEI according to the manufacturer’s protocol and all experiments were performed 72 h posttransfection. Last, to stimulate IR of COS-7^IR^ cells (overexpressing NEU-1) or HepG2 cells, insulin (Sigma-Aldrich, 100 nM) was added to the culture medium. After an incubation period of 30 min at 37 °C, protein extraction was realized.

### MTT assays

HepG2 cell line and COS-7^IR^ cells were, respectively, harvested in 96-well plates at a cell density of 80,000 cells/well and 10,000 cells/well. After 24 h, cells were incubated with IntPep-RKR or mutIntPep-RKR at 0.1 μM or only with LDS micelles during 3 h or 24 h. Then, the medium has been removed and cells were incubated in the dark during 4 h at 37 °C with a *3-(4-5-dimethylthiazol-2-yl) -2,5-diphenyltetrazolium bromide* solution (MTT, Sigma, 5 mg/ml) diluted in PBS (one-sixth v/v). The medium was removed, and 100 μl of dimethyl sulfoxide were added in each well. Cell viability was measured at 570 nm with Infinite F200 Pro (TECAN) hardware using Magellan software (https://lifesciences.tecan.com/software-magellan?p=tab--2).

### Immunofluorescence

HepG2 cells were seeded onto coverslips in 24-well plates at 50,000 cells/well. After 24 h, cells were fixed with paraformaldehyde 1% (Euromedex) for 5 min followed by a second incubation with paraformaldehyde 2% for 10 min at room temperature (RT). Cells were washed twice for 5 min with PBS and permeabilized with Triton 0.2% during 5 min at RT. A total of 3% bovine serum albumin (BSA) was disposed in each well for 1 h at RT after three washes with PBS for 5 min. Cells were then incubated with NEU-1 mouse monoclonal antibody (1/200 in PBS with 3% BSA) (Santa Cruz Biotechnology) for 2 h at RT. Then, this solution was removed, and the cells were incubated with anti-mouse secondary antibody coupled with Alexa Fluor 568 (1/1000 in PBS with 3% BSA) (Invitrogen) for 2 h at RT in the dark. After washes, cells were incubated with IntPeps coupled with FITC (0.1 μM) or with FITC alone (0.1 μM) at RT in the dark. Coverslips were then mounted in a medium containing 4′,6-diamidino-2-phenylindole (Prolong gold, antifade, Invitrogen). Slides were finally observed using confocal microscopy (Zeiss LSM 710) and featured slices were analyzed with Image J software (https://imagej.net/ij/download.html).

### NEU-1 dimerization

HepG2 cells grown in 75 cm^2^ flasks at 80% of confluence were washed twice with cold PBS and resuspended with 1 ml cold TEM buffer (75 mM Tris, 2 mM EDTA, 12 mM MgCl_2_, at pH 7,5) extemporaneously completed with 1% (v/v) protease inhibitor cocktail, 10 mM NaF, and 2 mM Na_3_VO_4_. After sonication, samples were centrifuged at 600*g* for 10 min at 4 °C to remove unlysed cells and nuclei. Supernatants were collected and centrifuged at 20,000*g* for 45 min at 4 °C. Crude membrane-containing pellets were then resuspended in TEM buffer supplemented with 1% (w/v) CHAPS {3-[(3-cholamidopropyl) dimethylammonio]-1-propanesulfonate} (Sigma-Aldrich). Cell membranes were then incubated with peptides at 0.1 μM and solubilized for 3 h at 4 °C under gentle end-over-end mixing. Supernatants were recovered after a centrifugation step (20,000*g*, 45 min, and 4 °C). These samples were then used for Western blot analyzes.

### Sialidase activity

HepG2 cells grown in 75 cm^2^ flasks and COS-7 cell lines grown in 10 cm Petri dishes were washed twice with cold PBS and resuspended with 1 ml cold TEM buffer extemporaneously completed with 1% (v/v) protease inhibitor cocktail, 10 mM NaF, 2 mM Na_3_VO_4_. After sonication, samples were centrifuged at 600*g* for 10 min at 4 °C to remove nonlysed cells and nuclei. Supernatants were extracted and centrifuged at 20,000*g* for 45 min at 4 °C. Crude membrane-containing pellets were then resuspended in 400 μl Mes buffer (2-(N-morpholino) ethanesulfonic acid hydrate, 20 mM, pH 4.5). Protein concentration was then determined by bicinchoninic acid assay to measure sialidase activity from 50 μg of crude membrane proteins. Samples were incubated with peptides for 15 min on ice. Muf-NANA (2′-(4-MethylUmbelliFeryl)-alpha-D-N-AcetylNeuraminic Acid) (Biosynth) at a concentration of 400 μM was then added and incubated with samples for 2 h at 37 °C in the dark. The reaction was then stopped by adding Na_2_CO_3_ (Merck). A total of 150 μl of each sample were finally transferred in black 96 well plates. Emitted fluorescence was measured with Infinite F200 Pro (TECAN) hardware and Magellan software (excitation: 360 nm/emission: 465 nm) (https://lifesciences.tecan.com/software-magellan?p=tab--2).

### Western blot

Protein samples were diluted in Laemmli buffer (500 mM Tris, 10% (w/v) SDS, 20% saccharose (w/v), 0.5% (w/v) bromophenol blue, 10% (v/v) β-mercaptoethanol, pH 6.8) and heated for 5 min at 100 °C. After electrophoresis in a 10% acrylamide SDS-PAGE gel with migration buffer (25 mM Tris, 192 mM glycine, and 0.1% SDS), proteins were transferred onto nitrocellulose membrane at 100 V for 1 h 30 min in transfer buffer (25 mM Tris, 192 mM glycine, and ethanol 20% (v/v)). Membranes were blocked for 1 h 30 min at RT with 0.1% TBS-Tween-20 (TBST) (50 mM Tris, 150 mM NaCl, pH 7.5, 0.1% (v/v) Tween) supplemented with 5% BSA. Membranes were incubated with anti-NEU-1 (Santa Cruz Biotechnology), anti-β-Actin (Santa Cruz Biotechnology), anti-IR (Cell Signaling Technology), anti-PPCA (Santa Cruz Biotechnology), anti p-IR (Y1185) (Abcam), anti-Akt (Cell Signaling Technology) or anti-p-Akt (S473) (Cell Signaling Technology) overnight at 4 °C. Membranes were then incubated at RT with corresponding horseradish peroxidase-linked or fluorescent probes-linked secondary antibody diluted in TBS-T for 1 h.

Chemiluminescent protein detection was done by using ECL Prime Kit (GE Healthcare, Orsay, France). Chemiluminescent and fluorescent protein detection was conducted using Odyssey Fc (LI-COR) hardware. Quantification of bands intensity has been made using Image Studio Lite software (https://www.licor.com/bio/image-studio/).

### Animal models

Experiments procedures were evaluated by research ethics committee of the University of Reims Champagne-Ardenne and approved by the French Ministry of Higher Education, Research and Innovation (APAFis #33891-2021111016547019). Five C57Bl/6J male and five female mice (Janvier Labs), aged 6 weeks, were maintained in a 12/12-h light/dark cycle and in a temperature and humidity-controlled environment. All mice had *ad libitum* access to a diet without chlorophyll (#U8959, SAFE diet) and water during the experimental period. Mice weight was measured twice per week throughout treatment. Weight gain or loss was then calculated and normalized to the weight measurement of the first week. The food intake was evaluated with weight feeders during the last week of treatment and every day. This weight was then divided by the number of mice in each cage.

### Animal treatments

Treatments have been started when mice were aged 8 weeks. Mice were under diet without chlorophyll (#U8959, SAFE diet) 2 weeks before the beginning of treatments to minimize the background noise of fluorescence measurements. LDS micelles or Cy5-IntPep-RKR were prepared as described previously and injected intraperitoneally at 100 μg/kg twice per week (100 μl).

### Plasma assays

Blood samples were collected before and after 8-weeks treatment, respectively, with LDS micelles or Cy5-IntPep-RKR by retroorbital and intracardiac pathway with sterile heparin Pasteur pipets and sterile needle in lithium heparin MiniCollect tubes (Greiner Bio-One). Animals were under isoflurane anesthesia during blood recovery. After centrifugation (3000 rpm, 10 min), plasma samples were then recovered in 1.5 ml eppendorfs and conserved at −20 °C. Finally, these samples were sent to the *Institut Clinique de la Souris* (Illkirch, France) to quantify several parameters as AST, CRP, and LDH. Graphics represent the percentages of observed variation between values obtained before and after 8-weeks treatment with LDS micelles or Cy5-IntPep-RKR.

### Biodistribution studies

Fluorescence measurements in entire body were realized in 13 mm cassette under isoflurane anesthesia (with PerkinElmer fluorescence molecular tomography 4000) at first, second, fifth, seventh, and eighth weeks of the treatment and 15 min after injection of fluorescent peptides. Organs were collected at the end of the treatment and placed on another cassette to measure their fluorescence with the same device. To remove the potential autofluorescence of certain organs, organ fluorescence of mice receiving LDS micelle injection (twice per week at 100 μg/kg) was measured and subtracted to those of mice receiving Cy5-IntPep-RKR. The measurement of organ fluorescence has been converted in ng of peptides per gram of organ and represented in % of peptides per organ. The software TrueQuant has been used for analyzes (https://www.perkinelmer.com.cn/lab-products-and-services/resources/in-vivo-imaging-software-downloads.html?_ga=2.29909951.1041849854.1637318373-1604795059.1637318373). The geometric size of the region of interest (ROI) background was between 40 and 41.5 mm^3^, and one of the ROI was between 7900 and 8100 mm^3^ in the entire body. The geometric size of the ROI background was the same in organ per organ biodistribution and one of the ROI for each organ approximatively as followed: the adipose tissue (1825 mm^3^), the spleen (1095 mm^3^), the stomach (2555 mm^3^), the bladder (594 mm^3^), the muscle (1142 mm^3^), the liver (3379 mm^3^), and the kidneys (1946 mm^3^).

### Glycemia measurement

A slight incision at tail was made to collect a drop of blood (<10 μl). The nonfasted blood glucose was then measured under anesthesia for all groups with Contour Plus device and reactive strips before injection and 30 min after injection of LDS micelles or Cy5-IntPep-RKR at 100 μg/kg. Data were then normalized for each group by comparison with glycemia value before injection.

### Statistical analyses

Results are expressed as mean ± SEM. Statistical significance was evaluated using Student’s *t* test with GraphPad Prism software (https://www.graphpad.com/features).

## Data availability

The original contributions presented in the study are included in the article/Supplementary Material, and further inquiries can be directed to the corresponding authors.

## Supporting information

This article contains supporting information.

## Conflict of interest

The authors declare that they have no conflicts of interest with the contents of this article.

## Figures and Tables

**Supporting Figure S9:**