# Supplementary material for: Inhibition of neuraminidase-1 sialidase activity by interfering peptides impairs insulin receptor activity in vitro and glucose homeostasis in vivo
Source: J Biol Chem. 2024 Apr 23;300(6):107316. doi: 10.1016/j.jbc.2024.107316 (PMC11167521; doi:10.1016/j.jbc.2024.107316)
Supplement: Supporting Figures [file mmc1.pdf]

Supplementary Figure 1: Effects of LDS micelles containing peptides (mutIntPep-RKR or IntPep-RKR) and LDS micelles alone on cell viability. HepG2 cells **(A,B)** and COS-7 cells overexpressing IR **(C,D)** were incubated with mutIntPep-RKR, IntPep-RKR (0.1 µm) or LDS micelles for 3h **(A,C)** or for 24h **(B,D)**. Results are normalized using the control (no peptide and no micelle) condition (n = 3-4; ns: non-significant; ANOVA).

Supplementary Figure 2: Size determination of LDS micelles and LDS micelles containing peptides (mutIntPep-RKR, IntPep-RKR or FITC-IntPep-RKR) by DLS. Each suspension of micelles was analyzed at a scattering angle of 173° in triplicate at 22°C, using Zetasizer Nano ZS equipment (Malvern Panalytical, UK). Water was used as a reference dispersing medium. Micelles sizes were expressed in volume (as the diameter of a theorical sphere in nanometer), by following the Malvern software instructions.

Supplementary Figure 3: Study of IR and NEU-1 expression in HepG2 cells **(A)** and IR, NEU-1 and PPCA **(B)** in COS-7 overexpressing IR by western-blot. The central panel of the Figure S3A has been reused in Figure 1A. The panel corresponding to n=3 of the Figure S3B has been reused in the Figure 1B.

Supplementary Figure 4: Colocalization between interfering peptides and membrane NEU-1. Localization of FITC-IntPep-RKR at 0,1 µm (green) and NEU-1 (red) in HepG2 cells. Colocalization between interfering peptides and NEU-1 are showed by white arrows (right panel) (n = 3).

Supplementary Figure 5: Colocalization between FITC and membrane NEU-1. Localization of FITC at 0,1 µm (green) and NEU-1 (red) in HepG2 cells (n = 3).

Supplementary Figure 6: Effects of interfering peptide on NEU-1 dimerization in HepG2 cells (n = 4). The top-left-side panel of the Figure S6 has been reused in Figure 2A.

Supplementary Figure 7: Effects of interfering peptide on IR and Akt phosphorylation in HepG2 cells. The top-right-side panel of the Figure S7 has been reused in Figure 4A.

Supplementary Figure 8: Effects of interfering peptide on IR phosphorylation in COS-7 cells overexpressing IR and NEU-1. The top-left-side panel of the Figure S8 has been reused in Figure 4D.

Supplementary Figure 9: Effects of LDS micelles and Cy5-IntPep-RKR at 100 µg/kg on liver and cellular toxicity and inflammation of C57Bl/6 mice before and after 8 weeks of treatment. Plasma collected before and after treatment were analyzed for AST **(A)**, CRP **(B)** and LDH **(C)** by the *Institut Clinique de la Souris*. Graphics represent the mean percentage of observed variation between values obtained before (untreated) and after treatment ± SEM. Displayed

significance corresponds to comparison between dosage values before (untreated) and after treatment (n = 10 per group, ns: non-significant; ***$p < 0.001$; ****$p < 0.0001$).
