# Supplementary figures and images for: Inhibition of neuraminidase-1 sialidase activity by interfering peptides impairs insulin receptor activity in vitro and glucose homeostasis in vivo
Source: J Biol Chem. 2024 Apr 23;300(6):107316. doi: 10.1016/j.jbc.2024.107316 (PMC11167521; doi:10.1016/j.jbc.2024.107316)

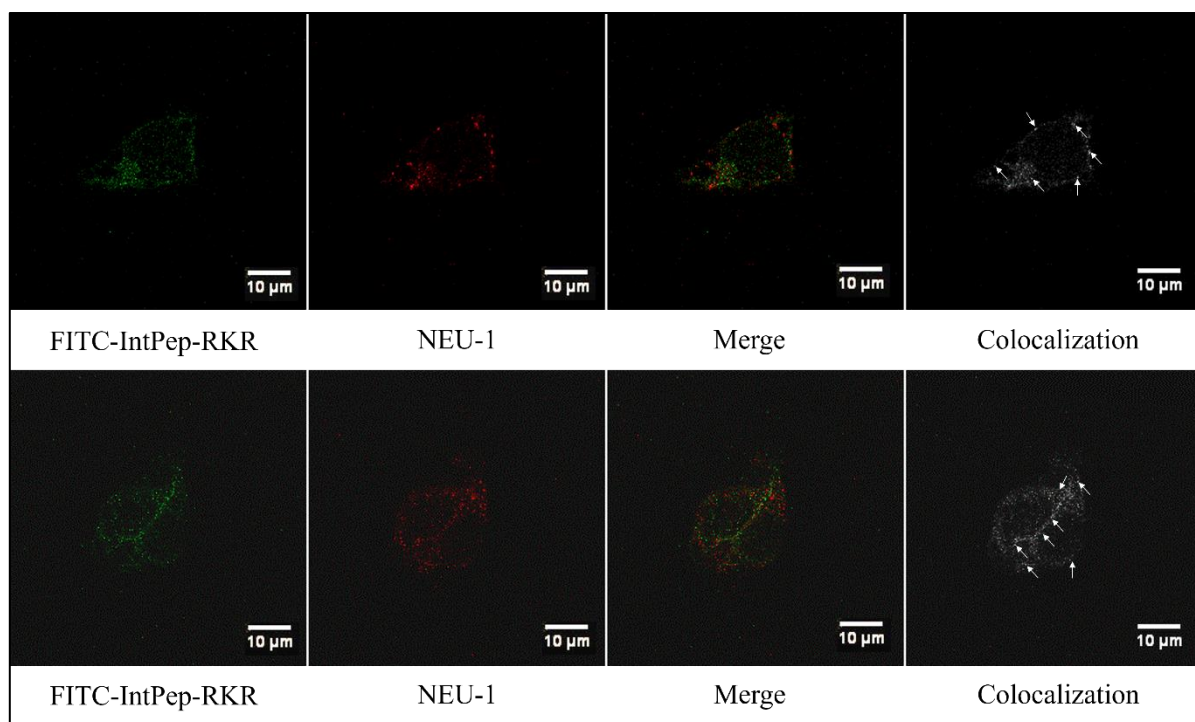

**Supplementary Figure 4:** Colocalization between interfering peptides and membrane NEU-1.

Supplement: Supporting Figure S3 [file mmc4.pdf]

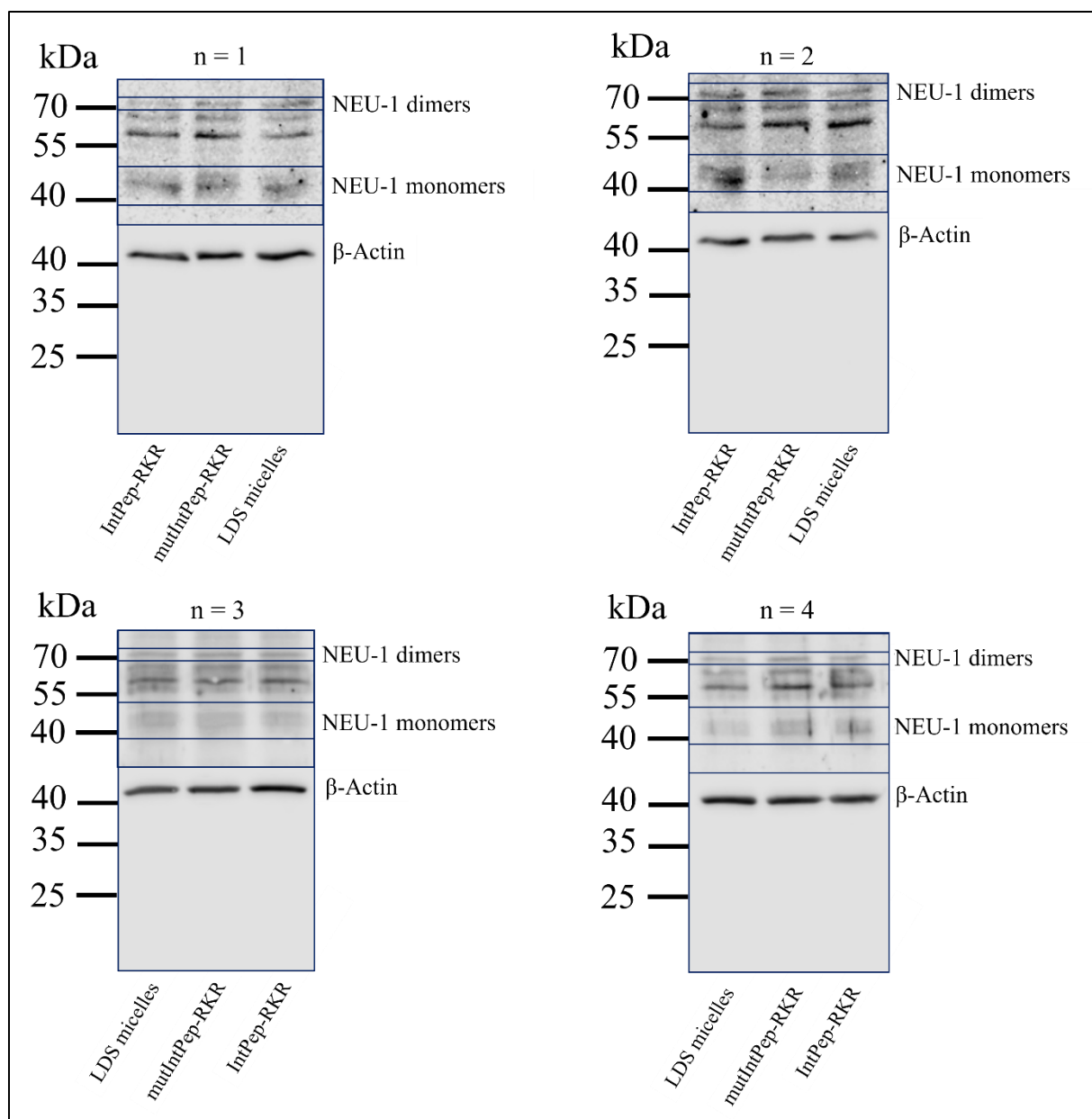

**Supplementary Figure 6:** Effects of interfering peptides on NEU-1 dimerization in HepG2 cells.

Supplement: Supporting Figure S5 [file mmc6.pdf]
