# Supplementary material for: Inhibition of neuraminidase-1 sialidase activity by interfering peptides impairs insulin receptor activity in vitro and glucose homeostasis in vivo
Source: J Biol Chem. 2024 Apr 23;300(6):107316. doi: 10.1016/j.jbc.2024.107316 (PMC11167521; doi:10.1016/j.jbc.2024.107316)
Supplement: Supporting Figure S4 [file mmc5.pdf]

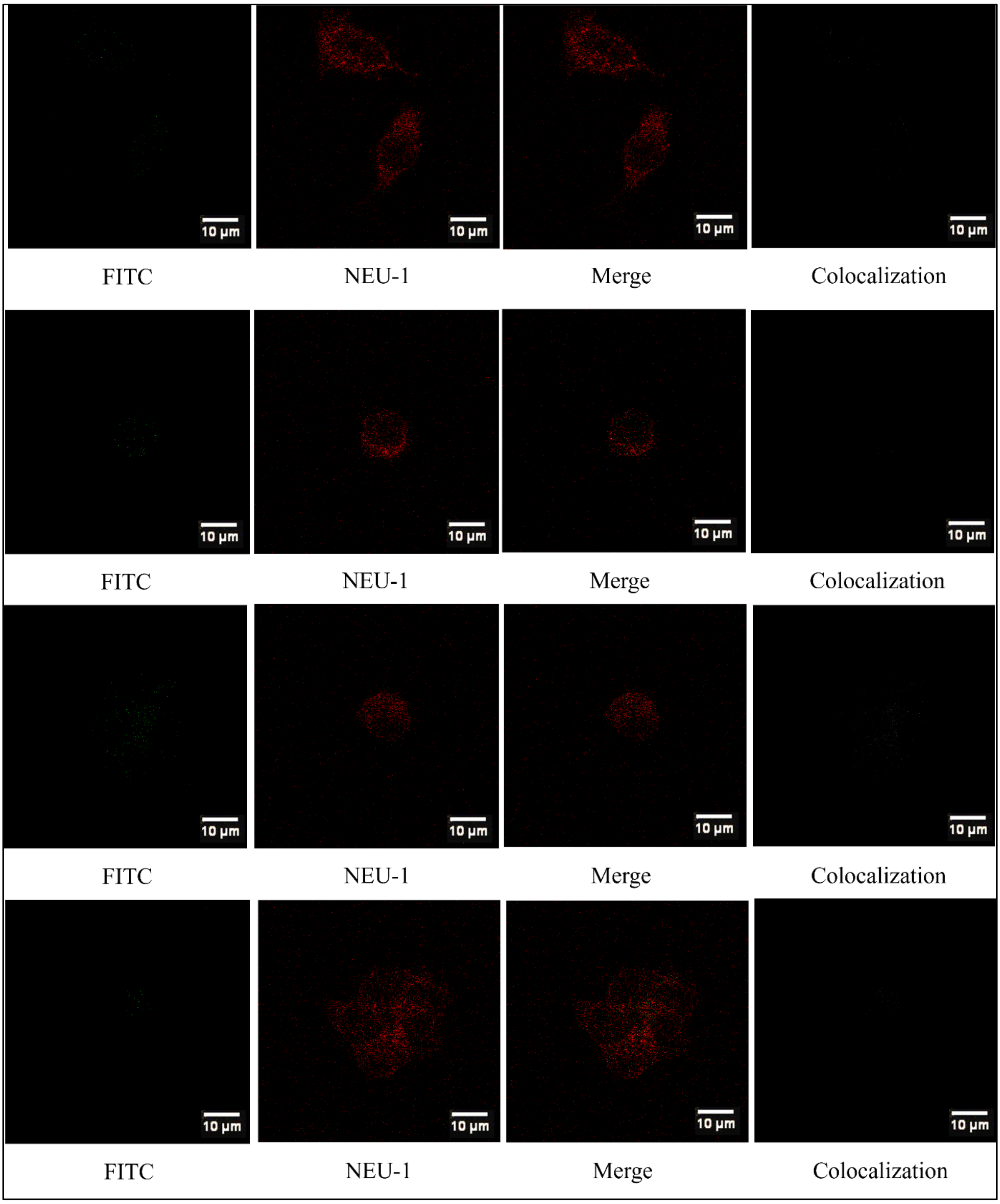


**Supplementary Figure 5:** Colocalization between FITC and membrane NEU-1. Localization of FITC at 0,1 µm (green) and NEU-1 (red) in HepG2 cells (n = 3).
