# Supplementary material for: Inhibition of neuraminidase-1 sialidase activity by interfering peptides impairs insulin receptor activity in vitro and glucose homeostasis in vivo
Source: J Biol Chem. 2024 Apr 23;300(6):107316. doi: 10.1016/j.jbc.2024.107316 (PMC11167521; doi:10.1016/j.jbc.2024.107316)
Supplement: Supporting Figure S7 [file mmc8.pdf]

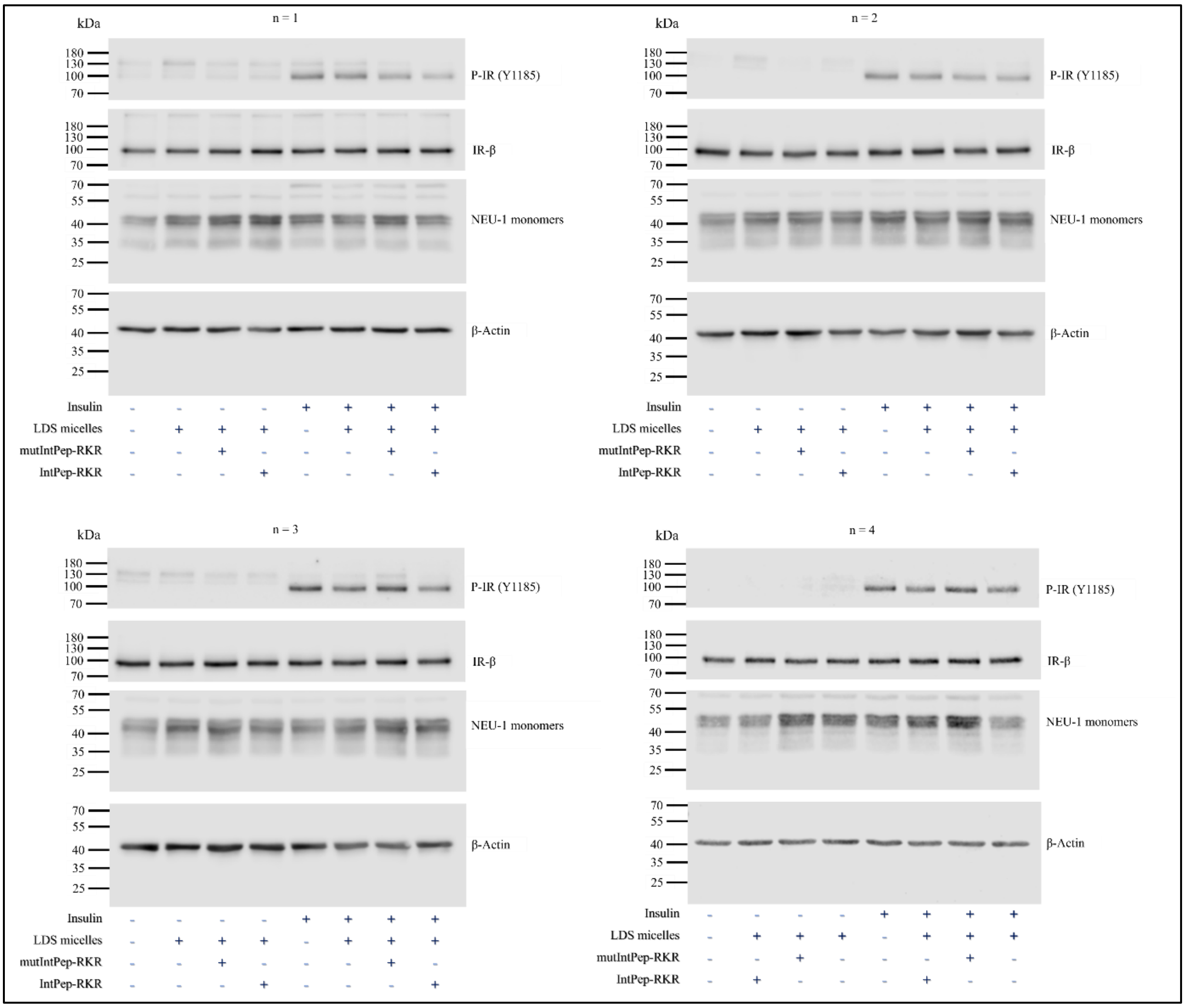

Supplementary Figure 8: Effects of interfering peptide on IR phosphorylation in COS-7 cells overexpressing IR and NEU-1.
